# Assessment of vaccination service delivery and quality: a cross-sectional survey of over 1300 health facilities from 29 districts in Sindh, Pakistan conducted between 2017–18

**DOI:** 10.1186/s12913-022-08098-9

**Published:** 2022-06-01

**Authors:** Danya Arif Siddiqi, Sara Abdullah, Vijay Kumar Dharma, Tasleem Khamisani, Mubarak Taighoon Shah, Hamidreza Setayesh, Aamir Javed Khan, Subhash Chandir

**Affiliations:** 1 IRD Global; 583 Orchard Road, #06-01 Forum, Singapore, 238884 Singapore; 2 Q58254430 IRD Pakistan, 4th Floor Woodcraft Building, Korangi Creek, Karachi, 75190 Pakistan; 3 Gavi, the Vaccine Alliance, Chemin du Pommier 40, 1218 Le Grand-Saconnex, Switzerland

**Keywords:** Health facility assessment, Immunization delivery quality, Immunization infrastructure, Immunization processes

## Abstract

**Background:**

Routine childhood immunization coverage in Pakistan remains sub-par, in part, due to suboptimal utilization of existing vaccination services. Quality of vaccine delivery can affect both supply and demand for immunization, but data for immunization center quality in Pakistan is sparse and in Sindh province in Southern Pakistan, no comprehensive health facility assessment has ever been conducted at a provincial level. We assessed health facilities, specifically immunization centers, and their associated health workers throughout the province to summarize quality of immunization centers.

**Methods:**

An exhaustive list of health facilities obtained from Sindh’s provincial government was included in our analysis, comprising a total of 1396 public, private, and public-private health facilities. We adapted a health facility and health worker assessment survey developed by BASICS and EPI-Sindh to record indicators pertaining to health facility infrastructure, processes and human resources. Using expert panel ranking, we developed critical criteria (the presence of a cold box/refrigerator, vaccinator and vaccination equipment at the immunization center) to indicate the bare minimum items required by immunization centers to vaccinate children. We also categorized other infrastructure, process, and human resource items to determine high, low and moderate function requirements to ascertain quality. We evaluated presence of critical criteria, calculated scores for high, moderate and low function requirements, and displayed frequencies of infrastructure, process and human resource indicators for all immunization centers across Sindh. We analyzed results at the division level and utilized a two-sample independent clustered t-test to test differences in average function requirement scores between facilities that met critical criteria and those that did not.

**Results:**

Out of the 1396 health facilities assessed across Sindh province from October 2017 to January 2018, 1236 (88.5%) were operational while 1209 (86.6%) offered vaccination services (immunization centers). Only 793 (65.6%; 793/1209) immunization centers met the critical criteria of having all the following items: vaccinator, a cold box or refrigerator and vaccine supplies. Of the 416 (34.4%; 416/1209) immunization centers that did not meet the critical criteria, most of the centers did not have a cold box or refrigerator (28.3%; 342/1209), followed by lack of vaccines (19.9%; 240/1209), and a vaccinator (13.0%; 157/1209). Of the 2153 healthcare workers interviewed, 1875 (87.1%) were vaccinators, of which 1745 (81.0%; 1745/2153) were male, and had an average of 12.4 years of schooling. A total of 1805 (96.3%; 1805/1875), 1655 (88.3%; 1655/1875) and 1387 (74.0%; 1387/1875) of the vaccinators were trained in vaccination, cold chain and inventory management respectively.

**Conclusion:**

One out of three immunization centers in Sindh lack the critical components essential for quality vaccination services. While the majority of health workers (>80%) were trained on vaccination and cold chain management, the proportion trained on inventory management was comparatively low. Our findings therefore suggest that suboptimal immunization center quality is partly due to inadequate infrastructure and inefficient processes contributed to an extent, by low levels of inventory management training among vaccinators. Our study presents critical research findings with high-impact policy implications for identifying and addressing gaps to improve vaccination uptake within a low-middle income country setting.

## Background

Childhood immunizations are highly cost-effective and are mostly offered free-of-cost to clients in most countries to reduce child morbidity and mortality. Yet, approximately 19·7 million children globally have not received the three recommended doses of diphtheria, tetanus and pertussis (DTP) vaccine [1]. In Pakistan, the Expanded Program on Immunization (EPI) was launched in 1978 (renamed as the Federal Directorate of Immunization in 2022) with the mandate to reduce child morbidity and mortality related to six vaccine-preventable diseases. However, despite free provision of vaccines through the EPI, immunization coverage for fully immunized children (defined as children aged 12–23 months, who have received one dose of Bacille Calmette Guérin (BCG), three doses of Pentavalent, pneumococcal vaccine (PCV) and oral polio vaccine (OPV), and one dose of measles vaccines) is only approximately 66%, pointing to suboptimal utilization of existing vaccination services [2–4]. Coverage rates vary significantly across the country’s four provinces, with Sindh province reporting 49% coverage for fully immunized children which is less than the national coverage [2]. In part, further improvements in coverage can occur by identifying gaps in the quality of vaccine delivery via detailed data collection on health facilities to ascertain components that may or may not function at a service delivery level [5].

Quality of vaccine service delivery can affect both supply and demand for immunization. Good quality vaccination services can increase service utilization (demand) via positive attitudes of health workers and reduced waiting times. Simultaneously, better vaccination service delivery can also improve provision (supply) by ensuring adequate availability of vaccines and proper cold-chain equipment. The latter, comprising of functional refrigerators and cold boxes is of particular importance to maintain optimal vaccine storage temperatures in order to ensure vaccine viability, potency, [6] and to reduce wastage [7–9]. Functional cold chain equipment is therefore key to preventing vaccine formula degradation, ensuring optimal immunological response among recipients and reducing the need for re-immunization in populations [10, 11].

Despite known advantages of improving vaccination service delivery, quality of vaccination centers is sub-par in many developing countries. Health facility studies in Cameroon, Ghana, Kenya, and Uganda have noted limited resources for cold-chain management characterized by the limited use of cold boxes and vaccines being stored outside of the recommended temperature range [9, 12]. Moreover, in India and Bangladesh, vaccination centers have been shown to commonly face a shortage of vaccine supplies, with only 15% of the facilities in Bangladesh having proper storage capacity for vaccines with widespread shortages of pneumococcal conjugate vaccine (PCV) and inactivated polio vaccine (IPV) doses [13, 14]. In Pakistan, studies investigating gaps in vaccination service delivery pointed to the need for improved governance, human resource capacity building, better facility-level services, and addressing community perceptions as key areas for improving EPI [15], while also underscoring barriers to effective supportive supervision [16] and poor immunization-related knowledge among health workers [17]. Despite these overarching findings, there is a dearth of studies from Pakistan investigating the quality of services at immunization centers making it extremely difficult to identify specific issues or quantify the quality of the vaccination services provided through the provincial EPI.

This study addresses this gap by conducting a cross-sectional survey throughout Sindh province to assess vaccine-related infrastructure and practices at health facilities and the various cadres of health care workers providing immunizations, to delineate critical areas of improvement.

## Methods

### Study design and participants

A cross-sectional health facility assessment survey was conducted for all health facilities in the province of Sindh, Pakistan, and their associated health workers. Sindh province, in the south of Pakistan, is home to almost 47.9 million people living in 8.6 million houses [18]. With a population density of 339.9 people/km^2^ the province is spread across 7 divisions and comprises 29 districts. The under-5 mortality for the province is 104 per 1000 live births (compared to the national average of 74/1000 live births) and overall vaccination coverage for routine EPI immunizations is 49% (compared to the national average of 66%) [2, 18, 19]. The Expanded Program on Immunization in Sindh (EPI-Sindh) delivers free vaccination services via established health facilities. At the facility level, the primary management of vaccination services lies with the assigned fixed-center and outreach vaccinators.

Our sampling frame consisted of an exhaustive list of the health facilities obtained from the provincial government, comprising public and private facilities as well as public-private partnerships. Private facilities not part of government records, were beyond the scope of this assessment. As were the health facilities located in Districts Dadu and Khairpur, managed by a not-for-profit organization. To obtain health worker data, we interviewed all health workers registered at a specific facility for performing vaccination services. These included vaccinators, lady health workers and visitors (LHW/LHV) and other facility staff. 

### Vaccination schedule

As per the recommended schedule at the time of the survey, BCG vaccine was given at 0–6 weeks of age; pentavalent (DTP, hepatitis B, and *Haemophilus influenzae* b) vaccine, pneumococcal vaccine and oral/injectable polio vaccine, at 6, 10, and 14 weeks of age; and measles vaccine at 9 and 15 months of age. In 2018 and 2019, the Rotavirus and Typhoid Conjugate vaccines were also added to the immunization schedule at 6 and 10 weeks, and 9 months of age, respectively.

### Data tools

We modified the monitoring tools developed by BASICS and EPI-Sindh to assess the quality of vaccination services, vaccine supply, cold chain regulation, and health worker experience to fit the Pakistani context and developed one comprehensive tool for health facility assessment [20].

The adapted final survey had four main sections, including Quality of Service, Immunization Processes, Cold Chain Processes and Health Worker Data. Health worker data contained information on vaccinators, LHWs/LHVs responsible for administering immunizations at facilities and outreach, and their relevant demographics and background. Each section in the tool included a list of coded questions/statements based on identifying the presence of specific processes or equipment and close-ended questions to capture additional process information and barriers for optimal vaccine delivery. The adapted tool was pilot tested to ensure validity and translated into the local language prior to deployment in the field. The permission for the survey was obtained from the District Health Office in each district and the provincial EPI office.

### Data collection

Data was collected using an Android Open Data Kit application. District and town field coordinators (DFC and TFC) were hired and trained on data collection and inspection techniques. Each DFC was accompanied by a TFC to every health facility in their district. The data collectors first checked whether the facility was operational i.e. was open for patients and provided any kind of health service (including but not limited to immunizations), and then assessed the presence or absence of vaccination program equipment and materials by observing and inspecting the health facility and their vaccine, stock, and outreach records. Items specifically examined included an operational refrigerator, immunization registers, electrical power connection, back-up generator, tables, chairs, shaded waiting area, temperature monitor, and a cold box for emergencies. If equipment such as a cold box was unavailable for inspection as it was being used for outreach, study staff revisited the facility later to re-verify its presence. The data collectors also examined the process of immunizations such as whether the sessions were orderly, if vaccines were being administered in shade, usage of damaged vaccines and maintenance of temperature records. To minimize missing data, study staff conducted on-spot checks after surveying the facilities to ensure all fields had been appropriately filled, in case any item was missing, it was filled prior to leaving the site. If the survey could not be completed within a single visit, the facility was revisited at a later time to fill in the remaining questions.

After the facility assessment, study staff approached the registered health workers at the facility, explained the purpose of the assessment, and obtained verbal consent to administer a short quantitative survey. All registered health workers at a facility were interviewed. Workers not present at the time of health facility visits were approached later and for a maximum of three attempts until they were reached. The time to complete each health facility assessment was between 20 and 30 minutes, and health worker interviews took between 8 and 12 minutes.

### Data management and analysis

Data collected through the Open Data Kit was uploaded onto a secure database server. Only those involved with the project had access to the data for research purposes. Each health facility and associated worker was assigned a unique identifier at the time of data collection. All collected data was imported into the statistical package STATA. Data fields were frozen or locked post data collection and prior to initiating data analysis, to prevent data manipulation. To maintain data quality and minimize data entry errors, the data collection software only allowed acceptable data value ranges and prompted data collectors with error messages in case of inconsistent values. Additional cleaning and editing of data for outliers and inconsistencies was performed on an ongoing basis during the data collection phase.

All variables collected during the survey were characterized as either ‘infrastructure’, ‘process’ or ‘human resource’ variables. Two experts, a local and an international, were purposefully selected based on their experience on vaccination service delivery to rank the importance of each variable for facilities providing vaccination services as either ‘critical’, ‘high’, ‘moderate’ or ‘low’. The critical criteria were then developed (using all variables that the experts had marked as critical) to indicate the bare minimum items a facility needed to immunize children given current standards. Items assigned to the high, moderate and low function categories were considered as “value-adding” items with decreasing order of preference. The breakdown of the variables into the four categories via expert panel consensus can be seen in Table 1.

**Table 1:** Breakdown of items according to function requirement categories

Critical Criteria (***n*** = 3) 1. Vaccinator Present^a^ 2. Cold box Present or a Refrigerator Present^a^ 3. No Vaccination Equipment Shortage^b^		
High Function Requirement (***n*** = 8)	Moderate Function Requirement (***n*** = 4)	Low Function Requirement (***n*** = 8)
*Infrastructure* 1. Access to operational refrigerator 2. Cold box in stock for emergencies 3. Table and chair present 4. Shaded waiting area^c^ 5. Electricity connection *Process* 6. Absence of damaged vaccines *Human Resource* 7. Vaccinator trained in vaccination 8. Vaccinator trained in cold chain	*Infrastructure* 1. Vaccine stock register 2. Temperature monitor *Process* 3. Temperature record 4. Updated defaulter list	*Infrastructure* 1. Standard Vaccine Carrier for outreach 2. Updated immunization charts 3. Operational backup generator *Process* 4. Immunizations carried out in shade^d^ 5. Orderly session flow 6. Updated outreach plan 7. Consistent stock with Stock Register 8. Absence of food/drink in the vaccine refrigerator

^a^ Infrastructure ^b^Process

^c^Area where caregivers were waiting for their turn (for child to be vaccinated) was covered with a shade

^d^Vaccines were being administered in shade

Each facility was first evaluated for its critical criteria. In addition, we also generated scores for high, moderate, and low function requirements. Each facility was assigned a score of 1 for every item in the high and low function requirements present in the facility, and a score of 2 for every item in the moderate function requirement present in the facility. Therefore, each facility could get a maximum score of 8 for each of the three categories, with a higher score denoting better quality services. Once all facilities in a division were assigned a score for each of the three categories, the average scores were generated for each division and compared. For vaccinator training, the score out of one for each relevant training was calculated by using the percentage of the total vaccinators trained within the division.

We conducted the analysis at the division level to display frequencies of the infrastructure, process, and human resource indicators before presenting the division score rankings. A two-sample independent clustered t-test with an alpha of 0·05 was utilized to test differences in average function requirement scores between facilities that met critical criteria and those that did not. Statistical analyses were performed with STATA’13 [21].

## Results

### Health facility characteristics

We assessed a total of 1396 facilities across the seven divisions of Sindh province between October 2, 2017 and January 20, 2018. Out of the 1396 facilities, 88.5% (1236/1396) were operational (were open for patients and provided any kind of health service) and 86.6% (1209/1396) were both operational and offered vaccination services (facilities providing vaccination services are referred to as ‘immunization centers’ from this point on) (Table 2).

**Table 2:** Basic indicators of health facilities across divisions in Sindh, Pakistan

Division	Karachi (*n* = 287)		Hyderabad (*n* = 195)		Sukkur (*n* = 115)		Bambhore (*n* = 144)		Mirpurkhas (*n* = 199)		Larkana (*n* = 277)		Shaheed Benazirabad (*n* = 179)		Total (*N* = 1396)	
Population [22]	16,051,521		7,026,335		5,538,555		3566.300		4,228,683		6,192,380		5282.277		47,886,051	
Number of Vaccinators and Lady Health Workers Interviewed	483		314		172		255		252		396		281		2153	
Health Workers per 100,000 people^a^	3.0		4.4		3.1		7.1		6.0		6.4		5.3		4.5	
Health facility type: n (%)																
Public	177	(61·7))	64	(32·8)	80	(69·6)	69	(47·9)	106	(53·2)	137	(49·5)	98	(54·8)	731	(52·4)
Public-Private Partnership	21	(7·3)	98	(50·3)	33	(28·7)	70	(48·6)	77	(38·7)	132	(47·7)	77	(44·1)	510	(36·5)
Private	89	(31·0)	33	(17·0)	2	(1·7)	5	(3·5)	16	(8·0)	8	(2·9)	2	(1·1)	155	(11·1)
Operational Facilities: n (%)	284	(99·0)	190	(97·4)	115	(100)	109	(75·7)	155	(77·9)	214	(77·3)	169	(94·4)	1236	(88·5)
Operational Facilities administering vaccines: n (%)	284	(99·0)	187	(95·9)	113	(98·2)	103	(71·5)	153	(76·9)	204	(73·6)	165	(92·2)	1209	(86·6)
Frequency of vaccination at facilities that administer vaccines: n (%) (*n* = 1209)																
Daily	253	(89·1)	163	(87·2)	103	(91·2)	79	(76·7)	147	(96·1)	158	(77·4)	153	(92·7)	1056	(87·3)
Twice a week	16	(5·6)	1	(0·5)	2	(1·8)	2	(1·9)	3	(2·0)	14	(6·9)	2	(1·2)	40	(3·3)
Once a week	13	(4·6)	6	(5·3)	6	(5·3)	18	(17·5)	1	(0·7)	31	(15·2)	7	(4·2)	80	(6·6)
Occasional	2	(0·7)	18	(9·6)	2	(1·8)	4	(3·9)	2	(1·3)	1	(0·5)	1	(0·6)	30	(2·5)
Children immunized/day at facilities administering vaccines: median (range) (*n* = 1209)	51	(0–280)	22	(0–669)	46	(5–216)	37	(0–180)	34	(0–215)	35	(0–349)	32	(1–963)	37	(0–963)

^a^ Calculated by using population estimates and number of health workers interviewed

Of all facilities evaluated, 52.4% (731/1396) were primarily public facilities, 36.5% (510/1396) were public-private partnerships and 11.1% (155/1396) were private facilities. Karachi division had the highest population, number of health facilities, operational immunization centers and median number of children immunized daily. Of all the immunization centers, 87.3% (1056/1209) offered vaccinations daily, with a median of 37 children vaccinated per day. The average number of children immunized per day across public, private, and public-private partnership facilities were 54.5, 45.2 and 39.5 respectively (not shown).

### Infrastructure

Sukkur and Shaheed Benazirabad divisions had on average two vaccinators per immunization center as compared to one vaccinator per immunization center in other five divisions (Table 3).

**Table 3:** Infrastructure and equipment of health facilities administering vaccines (immunization centers) across divisions in Sindh, Pakistan

Division	Karachi (*n* = 284)		Hyderabad (*n* = 187)		Sukkur (*n* = 113)		Bambhore (*n* = 103)		Mirpurkhas (*n* = 153)		Larkana (*n* = 204)		Shaheed Benazirabad (*n* = 165)		Total (*N* = 1209)	
**Critical Criteria**																
Number of personnel administering vaccines per center: mean (SD^a^)	1·4	(0·1)	1·6	(1·4)	2·4	(5·0)	1·4	(0·9)	1·4	(1·0)	1·4	(1·1)	2·0	(3·3)	1·6	(2·2)
Cold box or a refrigerator present: n (%)	258	(90·8)	184	(98·4)	108	(95·6)	92	(89·3)	151	(98·7)	182	(89·2)	160	(97·0)	1135	(93·9)
**High Function Requirements**																
Access to operational refrigerator: n (%)	250	(88·0)	183	(97·9)	105	(92·9)	86	(83·5)	148	(96·7)	163	(79·9)	153	(92·7)	1088	(90·0)
Cold box in stock for emergencies: n (%)	122	(43·0)	125	(66·8)	65	(57·5)	63	(61·2)	109	(71·2)	126	(61·8)	112	(67·9)	722	(59·7)
At least one table and chair: n (%)	279	(98·2)	185	(98·9)	107	(94·7)	97	(94·2)	150	(98·0)	201	(98·5)	164	(99·4)	1183	(97·9)
Shaded waiting area: n (%)	282	(99·3)	185	(98·9)	111	(98·2)	100	(97·1)	146	(95·4)	199	(97·6)	164	(99·4)	1187	(98·2)
Electricity: n (%)	262	(92·2)	177	(94·6)	90	(79·6)	84	(81·6)	140	(91·5)	148	(72·6)	131	(79·4)	1032	(85·4)
**Moderate Function Requirements**																
Vaccine stock registers: n (%)	244	(85·3)	179	(95·7)	106	(93·8)	94	(91·3)	148	(96·7)	177	(86·8)	156	(94·6)	1104	(91·3)
Temperature monitor: n (%)	228	(80·3)	179	(95·7)	96	(85·0)	84	(81·6)	144	(94·1)	146	(71·6)	135	(81·8)	1012	(83·7)
**Low Function Requirement**																
Updated immunization charts: n (%)	177	(62·3)	161	(86·1)	105	(92·9)	89	(86·4)	125	(81·7)	142	(69·6)	158	(95·8)	957	(79·2)
Vaccine carrier for outreach: n (%)	206	(72·5)	160	(85·6)	108	(95·6)	89	(86·4)	132	(86·3)	172	(84·3)	159	(96·4)	1026	(84·9)
Operational back-up generator: n (%)	88	(31·0)	66	(35·3)	22	(19·5)	26	(25·2)	47	(30·7)	30	(14·7)	39	(23·6)	318	(26·3)

^a^Standard Deviation (SD)

More than 90% of the immunization centers across all divisions had a cold box or refrigerator present. Among the high function requirements, only 59.7% (722/1209) of all immunization centers had a cold box for emergencies, and 85.4% (1032/1209) of the immunization centers had an electricity connection. For the moderate function requirements, 91.3% (1104/1209) of the immunization centers had vaccine stock registers, while 83.7% (1012/1209) had temperature monitors throughout the province. Among the low function requirements, 79.2% (957/1209) of the immunization centers had updated immunization charts, 84.9% (1026/1209) had vaccine carriers, and only 26.3% (318/1209) had backup generators. Karachi, as compared to other divisions, had the least number of vaccine carriers for outreach (72.5% (206/284)) and cold box for emergencies (43.0% (122/284)), updated immunization charts (62.3% (177/284)), vaccine stock registers (85.3% (244/286)) and one of the lowest number of personnel administering vaccines (1·4 health care worker per immunization center).

### Vaccination processes

Generally, there were no children waiting for immunization during the study staff’s visit except in Karachi division where the median number of children waiting was two (Table 4).

**Table 4:** Processes of health facilities administering vaccines (immunization centers) across divisions in Sindh, Pakistan

Division	Karachi (*n* = 284)		Hyderabad (*n* = 187)		Sukkur (*n* = 113)		Bambhore (*n* = 103)		Mirpurkhas (*n* = 153)		Larkana (*n* = 204)		Shaheed Benazirabad (*n* = 165)		Total (*N* = 1209)	
Children (< 2 yrs) waiting for vaccination^a^: median, (range)	2 (0–25)		0 (0–5)		0 (0–6)		0 (0–8)		0 (0–40)		0 (0–37)		0 (0–84)		0 (0–84)	
**Critical Criteria**																
Vaccination equipment shortage in last 6 months: n (%)																
Vaccines	7	(2·5)	20	(10·7)	49	(43·4)	21	(20·4)	9	(5·9)	15	(7·4)	16	(9·7)	137	(11·3)
Syringes	6	(2·1)	10	(5·4)	7	(6·2)	18	(17·5)	7	(4·6)	19	(9·3)	23	(13·9)	90	(7·4)
Diluents	3	(1·1)	2	(1·1)	1	(0·9)	11	(10·7)	4	(2·6)	4	(2·0)	12	(7·3)	37	(3·1)
Others	12	(4·2)	15	(8·0)	6	(5·3)	6	(5·8)	13	(8·5)	18	(8·9)	25	(15·2)	95	(7·9)
**High Function Requirements**																
Centers with damaged vaccines: n (%)																
Expired	0	(0·0)	0	(0·0)	0	(0·0)	0	(0·0)	0	(0·0)	0	(0·0)	3	(1·8)	3	(0·2)
Frozen	0	(0·0)	0	(0·0)	0	(0·0)	1	(1·0)	0	(0·0)	0	(0·0)	3	(1·8)	4	(0·3)
**Moderate Function Requirements**																
Complete temperature record: n (%)	209	(73·6)	173	(92·5)	96	(85·0)	79	(76·7)	138	(90·2)	137	(67·1)	140	(84·8)	972	(80·4)
Updated Defaulter lists: n (%)	196	(73·6)	165	(88·2)	105	(92·9)	91	(88·4)	133	(86·9)	174	(85·3)	153	(92·7)	1017	(84·1)
**Low Function Requirements**																
Immunization carried out in shade: n (%)	280	(98·6)	185	(98·9)	112	(99·1)	98	(95·2)	144	(94·1)	204	(100·0)	163	(98·8)	1186	(98·1)
Orderly session flow: n (%)	275	(96·8)	158	(84·5)	105	(92·9)	74	(71·8)	142	(92·8)	195	(95·6)	154	(93·3)	1103	(91·2)
Updated outreach plan: n (%)	134	(47·2)	133	(71·1)	101	(89·4)	87	(84·5)	128	(83·7)	163	(80·0)	151	(91·5)	897	(74·2)
Consistent stock with stock register: n (%)	145	(51·0)	156	(83·4)	105	(90·3)	83	(80·6)	136	(88·9)	154	(75·5)	136	(82·4)	912	(75·4)
Food/drink in vaccine refrigerator: n (%)	7	(2·5)	1	(0·5)	6	(5·3)	6	(5·8)	6	(3·9)	3	(1·5)	16	(9·7)	45	(3·7)
^a^At the time of survey team’s visit																

However, the range of waiting times within divisions was quite large, especially in Shaheed Benazirabad, with some immunization centers having up to 84 children queuing for vaccination. Among the critical criteria, 11.3% (137/1209) and 7.4% (90/1209) immunization centers had a shortage of vaccines and syringes respectively with the highest vaccine shortage being in Sukkur (43.4% (49/113)) and syringe shortage in Bambhore (17.5% (18/103)) divisions at the time of visit. Among the moderate function requirements, of the 1209 immunization centers, 80.4% (972/1209)) had a complete temperature record with Larkana division having the lowest number of immunization centers (67.1% (137/204)) with complete records. It is pertinent to note that while the Karachi division had the highest median number of children waiting for vaccination (2), this division had the least number of immunization centers with updated outreach plans (47.2% (134/284)), updated defaulters lists (69.0% (196/284)) and consistent stock reports (51.0% (145/284)) compared to provincial averages of 74.2% (897/1209), 84.1% (1017/1209), and 75.4% (912/1209), respectively.

### Human resources

Of the 2153 healthcare workers interviewed, 1745 (81·0%) were male and had a mean age of 42·7 years with an average of 12·4 years of schooling (Table 5). Across the divisions, 1875 (87·1%) health workers were vaccinators, 110 (5·1%) were LHWs and 41 (1·9%) were LHVs. Of the 1875 vaccinators, 1805 (96·3%) and 1655 (88·3%) had been trained in vaccination and cold-chain management, respectively.

**Table 5:** Healthcare worker demographics across divisions in Sindh, Pakistan

Division	Karachi (*n* = 483)		Hyderabad (*n* = 314)		Sukkur (*n* = 172)		Bambhore (*n* = 255)		Mirpurkhas (*n* = 252)		Larkana (*n* = 396)		Shaheed Benazirabad (*n* = 281)		Total (*N* = 2153)	
Age in years: mean (SD^a^)	43·1	(10·0)	43·5	(9·5)	39·8	(9·4)	42·1	(8·6)	41·4	(9·2)	43·5	(9·3)	43·5	(10·0)	42·7	(9·5)
Gender																
Male (%)	321	(66·5)	253	(80·6)	150	(87·2)	197	(77·2)	227	(90·0)	360	(90·9)	237	(84·3)	1745	(81·0)
Years of schooling: mean (SD^a^)	12·0	(3·2)	12·5	(1·9)	12.6	(2·0)	11·4	(1·7)	12·2	(1·7)	13·2	(2·6)	12·5	(2·2)	12·4	(2·4)
Designation: n(%)																
Vaccinator	405	(83·8)	285	(90·8)	162	(94·2)	195	(76·5)	216	(85·7)	379	(95·7)	233	(82·9)	1875	(87·1)
Lady Health Visitor	10	(2·1)	9	(2·9)	0	(0·0)	7	(2·8)	11	(4·4)	0	(0·0)	4	(1·4)	41	(1·9)
Lady Health Worker	39	(8·1)	7	(2·2)	1	(0·6)	39	(15·3)	1	(0·4)	4	(1·0)	19	(6·8)	110	(5·1)
Other	29	(6·0)	13	(4·1)	9	(5·2)	14	(5·5)	24	(9·5)	12	(3·0)	23	(8·2)	124	(5·8)
Years of experience: mean (SD^a^)	18·7	(10·4)	19·0	(10·9)	16.4	(12·3)	18·5	(9·4)	15·9	(10·6)	20·3	(10·6)	20·3	(11·3)	18·7	(10·8)
Vaccinators trained in: n (%)																
Vaccination	397	(98·0)	279	(97·9)	152	(93·8)	179	(91·8)	212	(98·2)	371	(97·9)	215	(92·3)	1805	(96·3)
Cold Chain	384	(94·8)	175	(61·4)	150	(92·6)	167	(85·6)	203	(94·0)	360	(95·0)	216	(92·7)	1655	(88·3)
Inventory	337	(83·2)	148	(51·9)	142	(87·7)	129	(66·2)	188	(87·0)	259	(68·3)	184	(79·0)	1387	(74·0)
None	6	(1·5)	5	(1·8)	7	(4·3)	15	(7·7)	2	(0·9)	5	(1·3)	16	(6·9)	56	(3·0)

^a^Standard Deviation (SD)

### Quality scores of vaccination services by divisions

Only 65.6% (793/1209) immunization centers met the critical criteria of having a vaccinator, a cold box or refrigerator, and vaccine supplies, with the highest number of immunization centers situated in Karachi (75.7% (215/284)) and the lowest in Sukkur (38.0% (43/113)) (Table 6).

**Table 6:** Critical Criteria and Function Requirement Scores of health facilities administering vaccines (immunization centers) across Divisions in Sindh, Pakistan

Division	Karachi (*n* = 284)		Hyderabad (*n* = 187)		Sukkur (*n* = 113)		Bambhore (*n* = 103)		Mirpurkhas (*n* = 153)		Larkana (*n* = 204)		Shaheed Benazirabad (*n* = 165)		Total (*N* = 1209)		*P*-value
Overall Functionality Scores- Facilities meeting Critical Criteria																	
Facilities meeting Critical Criteria (critical criteria score = 3) n (%)	215	(75·7)	132	(70·6)	43	(38·0)	57	(55·3)	111	(72·6)	140	(68·6)	95	(57·6)	793	(65·6)	–
Facilities not meeting Critical Criteria, (critical criteria score < 3) n (%)	69	(24·3)	55	(29·4)	70	(62·0)	46	(44·7)	42	(27·4)	64	(31·4)	70	(42·4)	416	(34·4)	–
# of critical criteria elements met among facilities not meeting critical criteria, mean (SD^a^)	1·9	(0·3)	1·8	(0·4)	1·9	(0·2)	1·8	(0·4)	1·9	(0·3)	1·8	(0·4)	1·9	(0·3)	1·9	(0·4)	–
Facilities not meeting critical criteria-Without vaccinator n (%)	31	(44·9)	23	(41·8)	19	(27·1)	17	(37·0)	21	(50·0)	18	(28·1)	28	(40·0)	157	(13.0)	–
Facilities not meeting critical criteria-Without cold box or Fridge, n (%)	43	(62·3)	52	(94·5)	65	(92·8)	35	(76·0)	40	(95·2)	42	(65·6)	65	(92·9)	342	(28.3)	–
Facilities not meeting critical criteria-With vaccination supply shortage, n (%)	19	(27·5)	38	(69·1)	50	(71·4)	28	(60·9)	22	(52·4)	37	(57·8)	46	(65·7)	240	(19.9)	–
Highest Function Requirement Score, mean (SD^a^) (maximum score = 8)																	
Facilities meeting Critical Criteria (critical criteria score = 3)	7·2	(0·6)	6·8	(0·5)	6·9	(0·7)	6·9	(0·8)	7·4	(0·6)	7·1	(0·8)	7·2	(0·7)	7·1	(0·7)	0.182
Facilities not meeting Critical Criteria, (critical criteria score < 3)	6·6	(0·9)	6·6	(0·8)	7·0	(0·7)	6·5	(1·2)	7·4	(0·6)	6·6	(1·0)	6·9	(0·8)	6·7	(0·9)	
Moderate Function Requirement Score, mean (SD^a^) (maximum score = 8)																	
Facilities meeting Critical Criteria (critical criteria score = 3)	6·7	(1·9)	7·6	(1·0)	7·4	(1·3)	6·9	(1·6)	7·3	(1·3)	6·6	(2·2)	7·2	(1·4)	7·0	(1·7)	0.252
Facilities not meeting Critical Criteria, (critical criteria score < 3)	4·5	(2·7)	7·0	(2·0)	7·0	(1·9)	6·6	(2·0)	7·5	(1·3)	5·4	(2·6)	6·9	(1·6)	6·3	(2·3)	
Low Function Requirement Score, mean (SD^a^) (maximum score = 8)																	
Facilities meeting Critical Criteria (critical criteria score = 3)	5·7	(1·3)	6·6	(1·0)	6·8	(0·8)	6·3	(1·2)	6·6	(1·0)	6·2	(1·0)	6·7	(1·0)	6·3	(1·2)	0.527
Facilities not meeting Critical Criteria, (critical criteria score < 3)	4·8	(1·5)	6·1	(1·4)	6·6	(1·3)	5·8	(1·5)	6·4	(1·0)	5·6	(1·5)	6·5	(0·9)	6·0	(1·4)	

^a^Standard Deviation (SD)

Scoring the presence of high, moderate and low function requirements among those meeting critical requirements reveals a provincial average score of 7·1, 7·0, and 6·3 respectively, out of a maximum score of eight. Immunization centers that did not meet the critical criteria had on average 1·9 of the three required items. Of the 416 immunization centers not meeting critical criteria, 28.3% (342/1209) did not have a cold box or a refrigerator, 19.9% (240/1209) had vaccine supply shortages, and 13.0% (157/1209) had no vaccinator. The average scores for high, moderate, and low function requirements are lower for immunization centers that do not meet critical criteria, dropping to 6·7, 6·3 and 6·0 respectively, though they are not significantly different (*p* > 0.05) from the scores for immunization centers meeting the critical criteria. Function requirement scores across divisions were fairly consistent except for Karachi’s low function requirement score, which was significantly lower compared to other divisions regardless of critical criteria status.

## Discussion

Our results show that one out of every three immunization centers in the Sindh province did not possess the critical components, i.e. the presence of a cold box/refrigerator, vaccinator and vaccination equipment, required for the provision of vaccination. These immunization centers were primarily missing a cold box or a refrigerator, followed by vaccine supplies and/or a vaccinator. Scores generated based on the presence of high, moderate and low function requirements further contributed to recognizing missing components essential for effective vaccination, resulting in decreased quality and coverage of vaccination services. Health worker demographics, on the other hand, indicated high levels of training on vaccination and cold chain management, but comparatively low levels of training on inventory management.

Our analysis shows that the proportion of immunization centers meeting critical criteria in a division follows a similar trend to the proportion of full immunization coverage (FIC) rates within the divisions. For instance, Karachi has the highest FIC (55·2%) [22] within the province. Our data shows it also has the highest percentage of immunization centers meeting the critical criteria (75·7%). In contrast, the Sukkur division has a considerably lower FIC coverage of 33·7% [22] and the lowest proportion of immunization centers meeting critical criteria (38·0%) as per our analysis. This finding is consistent with the literature as the quality of immunization centers is linked to the availability of vaccination personnel and equipment, where quality deteriorates due to a lack of vaccines and poor storage equipment, which in turn leads to reduced vaccination service utilization and consequently lower coverage rates [7–9, 13, 23, 24]. Moreover, poor cold chain practices have also been associated with reduced coverage and increased disease outbreaks, such as the measles outbreak in Cameroon, due to loss in vaccine efficacy and increased wastage [9, 11]. Likewise, Nawabshah district in Sindh province also experienced an incident of adverse event following immunization in 2018 as a result of administration of improperly stored Measles vaccines by the health workers [25, 26]. Prior research on Pakistan has specifically identified the country’s poor coverage outcomes due to a dearth of properly trained managers to supervise EPI planning and activities resulting in a shortage of staff at centers and present staff being fatigued and demotivated [15]. The study also points to the inadequate maintenance of cold chain and its negative impact on vaccine efficacy in part, due to frequent power outages and lack of electricity back up at many places, corroborated by our finding of very few (< 30%) functional back-up generators at immunization centers [15].

Within divisions, immunization centers failing the critical criteria as a whole also did worse on all three function requirements scale. The presence of basic infrastructure therefore also has an impact on vaccination optimization behaviours, with immunization centers investing less in the latter due to a lack of essential equipment and supplies, leading to a further reduction in quality. However, fulfilment of critical criteria comes with its own set of challenges as certain divisions such as Hyderabad had a higher percentage of immunization centers fulfilling critical criteria but performed worse on the low function requirement scale, as opposed to divisions like Sukkur where fewer immunization centers completed the critical criteria but had higher low function requirement scores. One explanation for the latter is that failures in critical criteria, which were a result of limited personnel and equipment, might have been compensated by increased attention to minor vaccination functions. Nevertheless, more research needs to be conducted on how immunization centers cope as a response to a shortage of critical elements to fully understand how these behaviors can be leveraged to improve immunization outcomes.

It is noteworthy that all divisions, regardless of their critical criteria status, had relatively high function requirement scores, indicating that all divisions met at least half of the function requirements within each category. The high function requirement scores combined with the low critical criteria scores indicate that major quality issues at these immunization centers can be tackled by either simply introducing one or more key elements such as a vaccinator, a cold box, a refrigerator, or a better vaccine supply chain. Research has shown that vaccinator shortage is a key barrier towards improving immunization coverage in developing countries while weak cold chain systems also adversely impact vaccination service delivery through affecting vaccine supply and availability [9, 27, 28]. We also found that infrastructure and process indicators fared poorly as compared to indicators pertaining to vaccinator education and training. However, it is pertinent to note that the process indicators are closely linked to the quality of vaccinator training; procedural practices such as maintaining a temperature record are associated with proper vaccine storage, therefore targeted health worker training can also provide an effective channel to improve certain process indicators within immunization centers.

Additionally, our results also highlight other indicators to be targeted in areas where critical criteria status has been met. One such example is Karachi, the major urban division in the province, which has a high number of immunization centers meeting critical criteria but ranks the lowest for low function requirements compared to rest of the divisions. As low function requirement consists of components pertaining to vaccine carriers, updated defaulters lists, and outreach plans, our results indicate insufficient outreach in the division, which could be a significant barrier in improving coverage outcomes, as outreach in both rural and urban areas has been shown to increase caregiver satisfaction and service utilization [24, 29, 30]. Studies in India also discovered that incomplete immunization was more prevalent in urban versus rural areas partially due to the increased mobilization of target population by health workers in rural areas, further indicating that a lack of outreach in urban areas can negatively affect immunization outcomes [31]. In addition to the need for strengthening outreach in Karachi Division, it is also of notable concern that Karachi has the lowest density of health workers. Shortage of health workers indicates that these problems are interlinked and increasing human resources is imperative to strengthening outreach campaigns.

Although the survey for this study was conducted in 2017–18, we find that many of our findings remain relevant even today, given that there is little indication of substantial policy changes within the local health system. One of the key developments within EPI Sindh since our survey was conducted is the recruitment of additional vaccinators in the province, particularly in Karachi, a welcome step by immunization stakeholders, also substantiated by our survey findings of shortage of workforce within Karachi Division. Our findings are also critical in the context of the COVID-19 pandemic, which has led to a significant decline in routine immunization globally [32], as well as in Pakistan. An analysis from Sindh province showed that one out of every two children missed their routine immunizations during the provincial COVID-19 lockdown [33]. Successfully maintaining immunizations in the aftermath of COVID-19 and conducting post COVID-19 catchup immunizations would depend on the extent to which pre-existing gaps in vaccination service delivery are addressed. Preliminary findings have shown that while routine immunization coverage rates have rebounded after the COVID-19 lockdowns in Sindh, the increase has largely been driven by outreach immunizations, while footfall at fixed immunization centers has persistently declined [34]. Addressing gaps in quality of vaccination services that existed even before the pandemic is essential to effectively recognize potential points of weakness, especially at fixed immunization centers, and strategically develop recovery plans to restore and sustain coverage in the aftermath of the pandemic.

We recognize that this assessment has the limitation of being conducted with government employees who may want to make facilities appear better than they are. We tried to minimize this risk by ensuring all facility equipment was surveyed via direct observation by data collectors instead of reported presence by government employees. Secondly, we acknowledge that the infrastructure and process indicators measured are only proxies for program performance. For example, the presence of vaccinator training does not necessarily ensure retention of taught information, just as the presence of an electrical connection does not mean available electricity at the immunization center. However, the variables used in the study allow an evaluation of the minimum threshold the vaccination program needs to meet, providing an insight into the current capacity of the immunization centers throughout the province. Our study findings can broadly be generalized across other provinces in Pakistan and other similar settings given this was a universal survey consisting of both public and private health facilities and all health workers in the province providing immunizations. Future research investigating the quality of vaccination service delivery could disaggregate quality measures by public and private facilities to gain a better understanding of how the two sectors could work hand in hand to improve service delivery.

One of the key strengths of our study is providing granular, division specific information to EPI and other expanded partners on the gaps existing in the vaccination system so that policies can be tailored precisely to address them. Other studies from within the local context have outlined several practical steps to address gaps such as; recruitment of additional vaccinators, rationalizing vaccinator allocation across immunization centers [15], refresher trainings to improve human resource, revamping immunization centers and making them child friendly [35], introducing systematic monitoring systems to improve vaccine stocks and reduce shortages and wastages [36], hiring of trained cold chain technicians and exploration of alternate solutions such as solar energy for better cold chain maintenance [15] as well as more overarching governance reforms to improve financing, policies and management of the immunization landscape [15]. Without being prescriptive in how the gaps should be addressed, the focus of this study was to delineate the key areas of concern that individual divisions and districts should focus on to improve overall vaccination services in the province.

## Conclusion

One out of three immunization centers in Sindh province lacked the essential components for immunizing children: a cold box/refrigerator, vaccinator, and vaccination supplies, an important finding as we observe that the proportion of FIC is directly correlated to the quality of vaccination services in an area. In order to get out of firefighting mode with continued outbreaks of poliovirus and measles, and ensure effective COVID-19 catch-up immunizations, we need to have a systematic approach to monitor the quality of vaccination service delivery for useful resource allocation and to improve the performance of vaccination services.

## Data Availability

The datasets used and/or analysed during the current study are available from the corresponding author on reasonable request.

## Abbreviations

BCG: Bacille Calmette Guérin
DTP: Diphtheria-Tetanus-Pertussis
LMIC: Low and Middle Income Countries
EPI: Expanded Programme on Immunization
FIC: Full Immunization Coverage
IRB: Institutional Review Board
DHHS: US Department of Health and Human Services
OHRP: Office for Human Research Protections
SD: Standard deviation
VPDs: Vaccine-Preventable Diseases
LHW: Lady Health Workers
LHV: Lady Health Visitors
DFC: District Field Coordinator
TFC: Town Field Coordinator
